# Hemi-Hypertrophy of Limbs and Internal Organs

**Published:** 1904-06-18

**Authors:** 


### HEMI-HYPERTROPHY OF LIMBS AND INTERNAL ORGANS.

Even minor instances of asymmetry as regards opposite sides of the body are not very common, and conspicuous examples of such inequality must be classed as decidedly rare. In one of the latter Dr. Robert Hutchison[1] has had the opportunity of making a post-mortem examination and has found that the asymmetry extended to the internal organs. The case was that of an infant who was noticed to have an abnormally large left arm and leg at birth and who died when aged four months from an attack of broncho-pneumonia. In the limbs the hypertrophy was apparently due to an increase of the soft tissues, the bones being unaffected. The circumference of the left forearm exceeded that of the right by 1½ inches, whilst in the calf the disparity was 2½ inches. There were one or two small nævi, but these were not confined to the left side. At the autopsy the exaggerated tissue in the left limbs was found to be due entirely to subcutaneous fat; there was no nævoid tissue and no vascular dilatation. The brain and the pineal and pituitary bodies were normal. But in the case of the paired organs those of the left side were relatively large. Thus the left kidney was double the weight of the right, and an even greater disparity was noted in the case both of the adrenals and the testicles. The heart and the thyroid were normal, and the liver presented the usual disproportion between right and left lobes. The case is a most interesting and important one. The absence of vascular dilatation and excess bears closely upon the suggestion that cases of hemi-hypertrophy are examples of overgrowth due to an abnormal nutrient supply following increased vascularity. Further, such an extreme bi-lateral asymmetry, affecting also the internal organs, can scarcely be explained on the "trophic" theory; it suggests rather some early disturbance of the normal process of development such for example as bi-lateral inequality in the segmentation of the ovum.

[1] Brit. Jour. of Children's Diseases, June, 1904.

